# Clusterin transduces Alzheimer-risk signals to amyloidogenesis

**DOI:** 10.1038/s41392-022-01157-x

**Published:** 2022-09-23

**Authors:** Xi Liu, Rongbo Che, Wenping Liang, Yun Zhang, Liyong Wu, Chao Han, Hong Lu, Weihong Song, Yili Wu, Zhe Wang

**Affiliations:** 1 The National Clinical Research Center for Geriatric Disease, Department of Neurology, Advanced Innovation Center for Human Brain Protection, Xuanwu Hospital, Capital Medical University, Beijing, PR China; 2 Department of Neurology, The First Affiliated Hospital of Zhengzhou University, Zhengzhou, Henan PR China; 3 Department of Psychiatry, Shandong Collaborative Innovation Center for Diagnosis, Treatment & Behavioral Interventions of Mental Disorders, Institute of Mental Health, Jining Medical University, Jining, Shandong PR China; 4 Oujiang Laboratory (Zhejiang Lab for Regenerative Medicine, Vision and Brain Health), Institute of Aging, Key Laboratory of Alzheimer’s Disease of Zhejiang Province, Zhejiang Provincial Clinical Research Center for Mental Disorders, School of Mental Health and The Affiliated Wenzhou Kangning Hospital, Wenzhou Medical University, Wenzhou, Zhejiang PR China

**Keywords:** Neurodevelopmental disorders, Molecular medicine

**Dear Editor**,

Deposition of amyloid-β (Aβ) to form neuritic plaque (NP) is the hallmark of Alzheimer’s Disease (AD). Major non-genetic risk factors such as ageing, stroke, diabetes and other conditions facilitate AD pathogenesis via unclear mechanisms. Furthermore, the mechanism underlying NP formation is unclear. Increasing Aβ causes NP in familial AD patients and in transgenic AD mice robustly expressing Aβ, but the NP formation requires long-term Aβ accumulation. Homogenates of AD brains seed NP nucleation in receiving brains, but the nature of the seeds and the endogenous seeds are unknown. Dysregulation of clusterin (CLU) has been implicated in AD pathogenesis. *CLU* gene contains several AD-associated intronic SNPs and its product clusterin (CLU) is increased in the brain tissues, cerebrospinal fluid (CSF), and plasma of AD patients may have anti-amyloidogenic roles, but *CLU* knockout significantly reduces NP by unknown mechanism^1^ (Extended note 1).

CLU is a secretory protein mostly synthesized in astrocytes in the brain, but is highly inducible in neurons by AD risk factors. In the brains of 3-month wild-type mice, CLU was detected with specific antibodies (Supplementary Fig. S1a) only in neurons in brain stem (Supplementary Fig. S1b). In aged and type-2 diabetic (insulin-receptor inhibition) model mice, CLU extensively accumulates in cortical neurons (Fig. 1a, b). In stroke (middle cerebral artery occlusion, MCAO), lactic-acid-treated (mimicking acidification upon stroke), hemorrhage, and Herpes simplex virus-infected model mouse brains, CLU was upregulated in neurons and extracellularly in affected regions. Upon neuroinflammation induced by intracerebral-ventricle (ICV)-injected lipopolysaccharide, CLU upregulation was only extracellular (Supplementary Fig. S1c–h). Thus, all major AD risk factors converge at CLU upregulation. CLU localizes to all NPs in AD mice (Supplementary Fig. S1i), but no glial CLU was detectable under any condition.

**Fig. 1:**
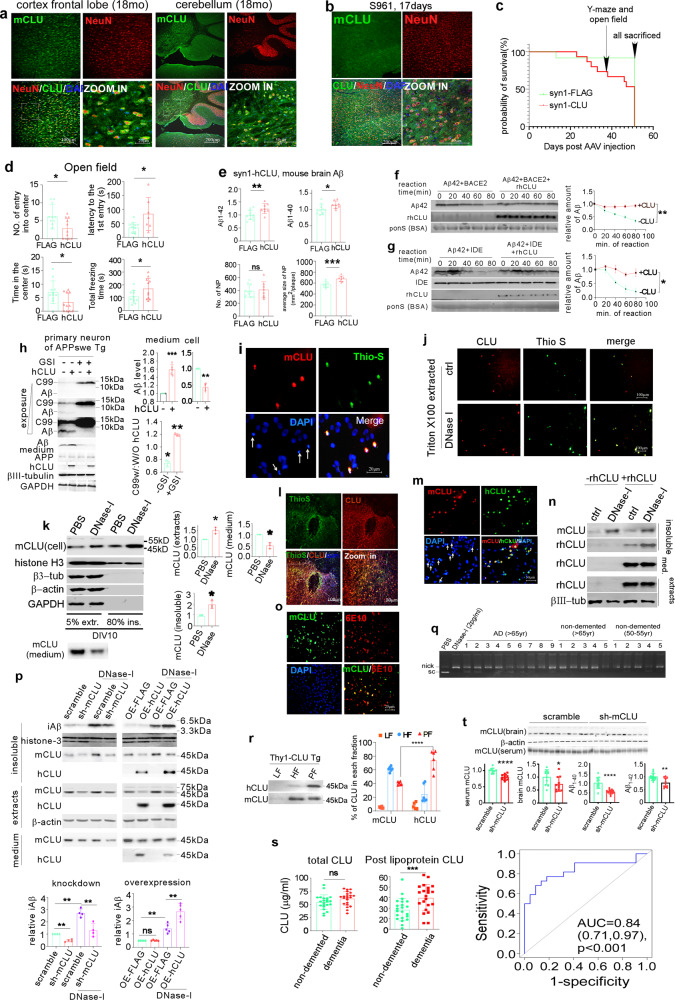
AD risks facilitate amyloidogenesis through CLU. **a** Representative images of brain slices of young (3mo) and aged (18 mo) mice co-stained with CLU and neuronal marker NeuN antibodies. **b** 3-month-old wild-type mice were ICV injected S961, an inhibitor against insulin receptor to mimic type-II diabetes. Brain slices of these mice 17d post injection were co-stained with CLU and NeuN. Neuronal CLU was not observed acutely (3d after injection) after S961 injection. Streptozotocin that reduces insulin to mimic type-I diabetes did not affect CLU even when the weight loss of mice was apparent (data not shown). **c** Survival curve of *APP/PS1ΔE9* mice after ICV injection of AAV overexpressing CLU. The time points of behavioral tests and the time of final mice sacrifice are indicated. In the Y-maze spatial memory test, half of the CLU-overexpressing mice did not make any alternation, and the experiment was inconclusive. **d** Open field assay of *APP/PS1ΔE9* mice overexpressing CLU or control in the brains. **e** Quantifications of Aβ1-40 and Aβ1-42 and the sizes and numbers of NP in *APP/PS1ΔE9* mice overexpressing CLU or control in the brains**. f**, **g** 300 nM synthetic Aβ_1-42_ was incubated with recombinant 80nM BACE2 (**f**) or IDE (**g**) in the presence and absence of 300nM recombinant hCLU (rCLU) for the indicated times, and indicated proteins were blotted for quantification. BSA added to the reaction was stained with Ponceau-S as internal standard. Aβ_1-42_ and CLU in human CSF are <2 ng/ml (~0.5 nM) and ~20 μg/ml (~280 nM), respectively, CLU is in much excess relative to Aβ_1-42_. **h** AAV9 expressing hCLU-FLAG or FLAG under *CAG* promoter were used to infect the primary neurons of *APPswe* transgenic (Tg) mice. The γ-secretase inhibitor L685,458 was added after differentiation on DIV4. Medium and intracellular Aβ and C99 were detected with 82E1 antibody. βIII-tubulin and GAPDH were used as internal standards for neurons and all cells, respectively. *n* = 5 repeats. **i** Primary neurons of wild-type mice were co-stained with mCLU, ThioS and DAPI for DNA. CLU in live neurons and contaminating astrocytes was below detectable level. Arrow: dead/dying cells indicated by condensed DNA staining. **j** Primary neurons were treated with PBS as control or 500U/ml DNase-I, and the cells were extracted with PBS containing 1% Triton X100 and the residual proteins on the coverslip were co-stained with mCLU antibody and thioflavin-S. **k** Primary neuron culture was extracted with 1% Triton-X100 buffer, and the indicated proteins in each fraction were blotted. The effects of DNase-I on endogenous mCLU in each fraction was quantified. extr.: Triton-X100 buffer extract, ins: Triton-X100 insoluble fraction. **l** 3-month-old wild-type mice were challenged with MCAO-induced stroke, and the brains were stained with ThioS and mCLU antibody 24 h post surgery. The images show the co-localization of CLU and ThioS (to indicate protein aggregates) in puncta or cells in the penumbra. *n* = 3. **m** Purified recombinant hCLU (rhCLU) was added to the primary neuron culture and the coverslip were co-stained with hCLU and mCLU antibodies. DAPI staining revealed the co-localization of hCLU, mCLU with condensed nuclei (arrows). **n** Purified rhCLU was added to the primary neuron culture, rhCLU in the extracts, insoluble fraction and conditioned media (med.) with or without overnight DNase-I treatment were blotted for rhCLU and endogenous mCLU in each fraction. **o** 1μM synthetic human Aβ_1-42_ was added to primary neuron culture, and after 3 days, the coverslips were stained with mCLU antibody and human APP/C99/Aβ specific antibody 6E10. **p** 1 μM synthetic Aβ_1-42_ was added to the primary neurons with knocked-down mCLU or overexpressed hCLU in the presence and absence of DNase-I. Triton-insoluble Aβ (iAβ) was blotted. *n* = 5 repeats. **q** Ageing and AD did not apparently modify the DNase activity of CSF. 30μl CSFs of indicated subjects were incubated with 1 μl pcDNA4-APP plasmid (60 ng/μl) for 24 h at 37 °C. Supercoiled (sc) and single strand broken (nick) plasmid DNA bands were separated on 1% agarose gel. Plasmid in PBS was used as a negative control, and 2 pg/ml DNase-I was added to a plasmid-PBS solution as a positive control. **r** serum hCLU and mCLU of the *Thy-1-hCLU* (clone #21) transgenic mice were fractionated by heparin-manganese precipitation. hCLU expressed in and secreted from the brain and mCLU in the LDL fraction (LF), HDL fraction (HF) and post-lipoprotein fraction (PF) were blotted and quantified. Compared to total endogenous mCLU in the serum, hCLU produced by neurons and secreted into the serum showed exceptionally high percentage in the PF fraction. **s** The sera of age- and sex-matched dementia patients (*n* = 22) and non-demented control (*n* = 22) were heparin-manganese fractionated. Total serum CLU and CLU in the PF fraction were ELISA quantified and compared between the two groups. The receiver operating characteristic (ROC) curves of PF CLU in predicting probability of dementia was plotted. **t** *APP/PS1ΔE9* mice were intraperitoneal injected with AAV8 expressing *shRNA* against *mCLU* (sh-mCLU, *n* = 8) or scramble control shRNA (*n* = 8). AAV8 was used to restrict the entry into brain parenchyma. Serum and brain mCLU were blotted and quantified. The levels of brain Aβ_1-40_ and Aβ_1-42_ were determined by ELISA (*n* = 11 scrambled shRNA, 11 mCLU shRNA)

Neuronal CLU is partly taken-up from extracellular space. Recombinant human CLU (rhCLU) appeared exclusively in cortical neurons upon ICV injection (Supplementary Fig. S2a). CLU is also endogenously expressed in primary neurons (PN). Several stress-inducers increased intracellular CLU, but only senescence by prolonged culturing^2^ upregulated both intracellular and extracellular CLU (Supplementary Fig. S2b, c). The extracellular increase was not simply due to accumulation over time, because medium CLU recovered overnight after complete medium change (Supplementary Fig. S2d). An *CLU*-shRNA driven by neuron-specific *syn1* promoter decreased CLU in a time-dependent manner (Supplementary Fig. S2e), further indicating an ageing-dependent CLU production in neurons. Therefore, CLU proteostasis in neuron is strictly regulated but changes during ageing. HDAC activator exifone and ATM kinase inhibitor Ku5933 inhibited CLU, suggesting epigenetics- and DNA damage-regulated CLU expression (Supplementary Fig. S2f, g).

To examine the effect of neuronal CLU upregulation on amyloidogenesis, 4.5-month *APP/PS1ΔE9* mice were infected by AAV-PHP.EB expressing human *CLU* (*hCLU*) under the *syn1* promoter by ICV injection. *hCLU* overexpressed under the astrocytic *GFAP* promoter in adult brains largely retained in astrocytes (Supplementary Fig. S3a), which is not a physiological/pathological condition. The overexpressed *hCLU* in neurons decreased the viability of *APP/PS1ΔE9* mice (Fig. 1c), and aggravated depression/anxiety (Fig. 1d) that are common symptoms of AD affecting cognition. *hCLU* expression increased Aβ_1-40_, Aβ_1-42_, and the average size of thioflavin-S (ThioS)-stained NPs by 14.81 ± 6.248%, 24.29 ± 8.781%, and 18.46 ± 4.182%, respectively (Fig. 1e, Supplementary Fig. S3b). Neurons with highly overexpressed hCLU showed decreased or abolished NeuN with concomitant active-caspase-3 (Supplementary Fig. S3c, d), suggesting apoptosis. Additionally, the A1 astrocyte marker complement-3 was upregulated in these mice (Supplementary Fig. S3e).

CLU enhances amyloidogenesis through multiple mechanisms. CLU at 1:1 ratio to Aβ_1-42_ abolished Aβ degradations by BACE2 and insulin-degrading-enzyme (Fig. 1f, g). (Extended note 2). Moreover, CLU strongly binds to the juxtamembrane-helix (JH) of C99 (Supplementary Fig. S4a),^3^ and competes for a key γ-secretase component nicastrin binding to C99, which lifts γ-secretase inhibition by JH^4^ and results in lower C99 level and higher extracellular Aβ^3^ in PC12 cell and in the PN of *APP* transgenic mice (Supplementary Fig. S4b–d, Fig. 1h). γ-secretase inhibition abolished CLU’s effect on C99 in PC12, but increased C99 in CLU-overexpressing PN compared to FLAG-expressing PN (Fig. 1h), presumably because CLU activates C99 generation via BACE2^3^ that is much higher in PN than in cell lines (Supplementary Fig. S4e). CLU overexpression reduced intracellular Aβ in PN (Fig. 1h) likely by enhancing Aβ secretion. γ-cleavage of Notch was unaffected by CLU (Supplementary Fig. S4f).

CLU in wild-type PN culture resides in extracellular puncta (Fig. 1i) resistant to Triton-X100 (TX) extraction and stained by ThioS (Fig. 1j, k). These puncta co-stained with condensed DNA indicating apoptotic/necrotic cells. ThioS and CLU co-stained puncta were also observed in penumbra after stroke (Fig. 1l). Both rhCLU and synthetic Aβ_1-42_ added to PN attached to these puncta (Fig. 1m–p). Mass spectrometry analysis revealed that all the TX-insoluble proteins are NP-enriched proteins despite of the absence of Aβ (Supplementary Table S1). These Aβ-independent puncta are therefore dubbed “NP seeds”.

To facilitate apoptotic cell clearance and prevent autoimmunity, CLU targets to apoptotic neutrophils through binding to surface histones.^5^ Apoptosis or oncosis of wild-type PN markedly increased TX-insoluble histone-H3 (Supplementary Fig. S5). DNase-I treatment to expose histones^5^ increased TX-insoluble endogenous mouse CLU (mCLU) and TX-insoluble rhCLU (Fig. 1k, n), suggesting stronger CLU attachment. Upon the addition of Aβ_1-42_ to PN, DNase-I treatment increased TX-insoluble Aβ_1-42_ (iAβ) by 1.5–2-fold. Suppressing *mCLU* in PN reduced iAβ by 47.56 ± 5.114% and 54.02 ± 4.957% with or without DNase-I treatment, respectively. Overexpressed hCLU in PN barely affected iAβ possibly because of sufficient endogenous mCLU, but enhanced DNase-I-dependent iAβ by 94.11 ± 11.34% (Fig. 1p). Hence, CLU is indispensable for iAβ deposition and acts synergistically with DNase to increase iAβ at the sites of cell death. Similar DNase activity has been detected in CSFs of AD and healthy people (Fig. 1q). Given that regardless of its effects on Aβ level (Extended note 3), CLU is required for NP in vivo,^1^ dead/dying cells may efficiently sequester soluble Aβ through CLU to generate NP, and Aβ level may be unessential (Supplementary Fig. S6).

CLU in the serum, CSF, and the conditioned medium of PN shows different pattern in heparin-manganese fractionation to isolate lipoproteins (Supplementary Fig. S7a, b). In a transgenic mouse where *hCLU* is expressed by the neuron-preferring *Thy-1* promoter (*Thy-1-hCLU*), hCLU is at a level ~1.3% of endogenous mCLU, ideal for metabolism investigations (Supplementary Fig. S7c, d). The neuron-expressed hCLU in sera displayed a high percentage in the post-lipoprotein fraction (PF) compared to mCLU (Fig. 1r), which prompted us to compared PF CLU in age- and gender-matched demented and non-demented people (Supplementary Table S2). PF CLU in the dementia group was significantly higher than in the non-demented group. PF CLU was of good discrimination with an AUROC of 0.84 (95% CI: 0.71, 0.97), a sensitivity of 77.3% (95% CI: 59.8%, 94.8%) and specificity of 81.8% (95% CI: 65.7%, 97.9). There was no difference between the same two groups when total serum CLU was tested (Fig. 1s).

While pro-amyloidogenic, CLU is also anti-amyloidogenic by enhancing Aβ excretion to the periphery.^1^ When AAV8 expressing *shRNA* against *mCLU* was intraperitoneally injected into *Thy1-hCLU* mice to peripherally inhibit *mCLU*, hCLU in brains were significantly reduced 3-weeks post injection (Supplementary Fig. S7e). In AD mice, brain and serum mCLU was mildly suppressed by the shRNA, and brain Aβ_1-40_ and Aβ_1-42_ were reduced by 45.93 ± 9.236% and 23.76 ± 6.693%, respectively, 7 weeks post injection (Fig. 1t). (Extended note 4).

Together, our study demonstrated that major AD risk factors converge at CLU upregulation. CLU inhibits Aβ degradation and promotes Aβ generation. Uncleared dead/dying cells upon AD risks seed insoluble Aβ deposition through histone-CLU-Aβ axis. The pro- and anti-amyloidogenesis functions of CLU should be considered for clinical applications (Supplementary Fig. S8). (Extended Discussion).

## Supplementary information

Supplementary information

Supplementary table S1

## Data Availability

The raw data are available from the corresponding authors.
