# Supplementary material for: Clusterin transduces Alzheimer-risk signals to amyloidogenesis
Source: Signal Transduct Target Ther. 2022 Sep 23;7:325. doi: 10.1038/s41392-022-01157-x (PMC9499966; doi:10.1038/s41392-022-01157-x)
Supplement: Supplementary file 1 — Supplementary information [file 41392_2022_1157_MOESM1_ESM.docx]

Supplementary Materials for

**Clusterin transduces Alzheimer-risk signals to** **amyloidogenesis**

Xi Liu^1,3*^, Rongbo Che^2*^, Wenping Liang^1*^, Yun Zhang^1*^, Liyong Wu^1*^, Chao Han^1^, Hong Lu^3^, Weihong Song^1,2,4#^, Yili Wu^2,4#^, Zhe Wang^1#^

Correspondence to Weihong Song [weihong@wmu.edu.cn](mailto:weihong@wmu.edu.cn); Yili Wu, Email: [wuyili@wmu.edu.cn](mailto:wuyili@wmu.edu.cn) and Zhe Wang, Email: [wangz@xwhosp.org](mailto:wangz@xwhosp.org)

**This PDF file includes:**

Materials and Methods

Supplementary Figures. S1 to S8, and table S2

Extended Note 1-4

Extended Discussion

**Materials and Methods**

**Study approval and ethics.** This study was approved by the Ethics Committees of the Xuanwu Hospital of Capital Medical University ([2020]026) and Guangdong Provincial People’s Hospital (KY-Z-2020-135-02, GDREC2018338H [R1]) China, and was conducted in accordance with the principles stated in the Declaration of Helsinki. Written informed consent was obtained from all the participants or legal guardians. Animal experiments were carried out in strict accordance with the guidelines for the Care and Use of Laboratory Animals in preclinical research from the Zhengzhou University, Jining Medical University, and Beijing Friendship Hospital Ethics Committees. The animal protocol was approved by the Institutional Animal Care and Use Committees of Zhengzhou University, Jining Medical University, and Beijing Friendship Hospital. All efforts were made to minimize the number of mice used and any potential suffering.

**Mouse strains and housing.** The male wildtype C57BL/6J mice (Stock No: 000664, young: weight,18-25g, 2~3 months; aged: weight, 24-35g, 16-18 months) or the transgenic mice carrying the *APPswe/PS1ΔE9* mutations (Stock No. [005864](https://www.jax.org/strain/005864), young: weight, 17-26g, 3~4 months; aged: 22-33g, 14-15 months) with C57BL/6J background were purchased from the Jackson laboratory. *APPswe* single transgenic mice (Stock No. C001076) were purchase from Cyagen Biosciences Inc. All mice were housed under automatic temperature- and humidity- controlled specific pathogen-free facility with 12-h light/dark cycle and *ad libitum* access to food and water.

***hCLU* transgenic mice**. For the generation of *Thy-1-hCLU* transgenic mice, cDNA coding for hCLU with a FLAG tag fused to the C-terminus was cloned into Thy1 1.2 expression vector to express CLU under the Thy1 promoter. The plasmid was linearized by EcoRI and PvuI, and microinjected into fertilized oocytes (performed by Cyagen Biosciences Inc). The survivors were transferred into pseudo‐pregnant recipient female mice. Surviving microinjected zygotes were implanted for potential founder pups. The founder pups were screened by genotyping using primers targeting *hCLU* mRNA. The transgenic mice were crossed until all the offspring were positive of hCLU to generate homozygous transgenic mice. For the generation of *CAG-hCLU* transgenic mice, cDNA coding for hCLU with a C-terminally fused FLAG tag under the control of the ubiquitous *CAG* promoter was inserted into the *ROSA* site.

**Mouse brain intracerebral stereotactic injection**. Mice were anaesthetized by intraperitoneal (i.p.) injection of a mixed solution of ketamine (100 mg/kg) and xylazine (10 mg/kg) in saline. The head was secured in a stereotaxic frame (RWD, Shenzhen, China). A 1cm longitudinal incision was made in the scalp to expose the skull, the x, y, z axis coordinates were all set to zero according to bregma. A burr hole was drilled and the Hamilton 7000 series syringe needle was placed as bregma coordinates (lateral ventricle: x:0.5 mm, y:0.8mm, z: -2.7mm; hippocampus: x:1.0mm, y: -1.7mm, z:-1.8mm; striatum: x: 1.5mm, y: 0.8mm, z:-3.0mm) relative to bregma. For ICH animal model construction, 0.75U collagenase VII-S (Sigma-Aldrich, St. Louis, MO) dissolved in 0.5μL saline was injected at a rate of 0.2μL/min into the mouse striatum. For *CLU* gene expression in the neuron in the brain, a total of 1×10^10^VG AAV-PHP.eb-Synapsin1-Clusterin or equal dosage of AAV-PHP.eb-Synapsin1-FLAG was injected into the bilateral ventricles. For clusterin gene expression in the astrocyte in the brain, 1×10^10^VG of AAV-PHP.eb-GFAP-Clusterin or equal dosage of AAV-PHP.eb-GFAP-mCherry was injected as the same procedure. For HSV-1 infection, 1×10^7^ VG HSV-1 was injected into the left hippocampus. For stress inductions, S961, LPS, Streptozotocin or lactic acid were delivered at designated dosage into the brain via the lateral ventricle or striatum. The needle was left in place for 10 min and then removed at a rate of 1 mm/min to prevent the reflux. After injection, the burr hole was filled with bone wax, the scalp incision was closed with cyanoacrylate glue (3M). During the operation procedure, body temperature was maintained at 37±0.5°C by an electronic thermostat-controlled warming pad (RWD, Shenzhen, China) until animals completely recovered from anesthesia and displayed normal motor activity. Successful ICH mouse model construction was defined as hemiplagia 6 hours after the operation. Animals were returned to their home cages, they were monitored closely over the next 4 h and then daily for the rest of the study.

**Intraperitoneal injection**. The 8-month-old *Thy-1hCLU* transgenic mice (#21) received one dose of 5×10^11^VG AAV8 expressing shRNA against *mCLU* or scrambled shRNA. Each of the 4-month-old *APP/PS1ΔE9* mice was injected with 1.5×10^12^ VG AAV8 expressing shRNA against *mCLU* or scrambled shRNA twice with a 3-weeks interval.

**Middle cerebral artery occlusion (MCAO)**. Investigators who were blinded to experimental grouping performed surgery of MCAO. Transient cerebral ischemia was induced by intraluminal occlusion of the left middle cerebral artery for 60 minutes as described previously with slight modifications^1^. Briefly, anesthesia was carried out by i.p. injection of a mixed solution of ketamine (100 mg/kg) and xylazine (10 mg/kg) in saline. Body temperature was maintained at 37°±0.5°C throughout the surgical procedure with a warming pad. The right common carotid artery, external carotid artery (ECA), and internal carotid artery (ICA) were exposed through a ventral midline incision. A round-tip, silicone coated 6-0 nylon filament was gently advanced through the lumen of the ICA to the opening of the middle cerebral artery (MCA). Successful MCAO was defined as a decrease in cerebral blood flow (CBF) of more than 80% compared with contralateral hemisphere, which was determined by laser-Doppler flowmetry (Moor Instruments, UK) monitoring. One hour after MCAO, the filaments were withdrawn to resume blood flow reperfusion. Animals were returned to their home cages, and they were monitored closely over the next 4 h and then daily for the rest of the study.

**Immunofluorescence.** Animals were euthanatized by deep anaesthetized with inhalation of isofluran, and transcardinal perfused with ice-cold PBS followed by 4% paraformaldehyde. Dissected brains were post-fixed in 4% paraformaldehyde at 4 °C overnight, followed by cryoprotection in sequential sucrose solution (15%-30%) for gradient dehydration. For immunofluorescence microscopy, mouse brains were embedded in O.C.T. compound (Tissue-Tek) and sections of 20μm thickness were prepared with a Leica cryostat. Primary antibodies were detected by appropriate secondary antibodies conjugated with fluorophore (Invitrogen). Images were captured by a Leica SP8 laser confocal microscope equipped with LAS software. Images were processed and quantified by Image J software (NIH) by an investigator blind to the experimental cohort. For NP staining by Thioflavin-S, the brain sections were dried on the glass slide and stained with thioflavin. NPs (cut off = 100 μm^2^) were quantified as previously described^2^. Images of stained brain sections were captured and analyzed by an investigator blind to the experimental cohort.

**Behavioral tests.** The open field test (OFT) was adopted to analysis spontaneous activity, anxiety-like behavior, and emotional change in the animals. 1day after OFT, Y-maze tests were performed to measure spatial learning memory and cognitive flexibility function of the mice. For the OFT , performed as previous reported literature^3^. Briefly, mice were placed in the corner of a plastic box (45 cm × 45 cm × 45 cm) and could move freely. The field was divided into 16 equal squares. The times spent in the peripheral zone, center zone and average speed were monitored and recorded for 5 min. The bottom board was wiped with 75% ethyl alcohol after each test. For the Y-maze test, mice were placed in the Y-shaped maze which consists of a three-armed chamber with the arms at a 120° angle apart from each other. Each arm is 35 cm long, 5.0 cm wide, and 10 cm high. Y-maze testing was conducted as reported previously^4^. The mice were placed in the center zone of the Y-maze, and arm entries were monitored and recorded for 5 min. Spontaneous alternation behavior was defined as consecutive entries into all three arms without repeated entries and was expressed as spontaneous alternation percentage (SAP) of the total arm entries. Video footage was captured by a Sony camera and analysis by the Any-maze (Stoelting Co.,USA) software. All behavioral tests were carried out and data analysed by independent investigators double-blind to the experimental design and animal grouping.

**Cell culture and transfection**. HEK293 and SH-SY5Y cells were cultured in Dulbecco’s modified Eagle’s medium (DMEM) containing 10% fetal bovine serum (FBS). PC12 cells were cultured in DMEM supplemented with 15% FBS. All cells were maintained at 37°C in an incubator containing 5% CO_2_. Plasmids were transfected into cells with lipofectamine 2000 or Polyethylenimine (Linear, MW 25,000). To detect Aβ generated in HEK293 cells, complete medium was changed on the second day after transfection with FBS-free DMEM and maintained for 24 hours before harvest.

**Primary mouse cortical neuron culture**. Cortical neuron were prepared from E18 mouse embryos as previously described^2^. For shRNA-mediated knockdown and overexpression, 1 × 10^11^ genomic copies (GC) of AAV9 were added to each well of 12-well plates on day 3 *in vitro* (DIV3) and the media were changed on DIV 4. For drug treatments, the drugs were added to cells at the indicated time. For prolonged culture, half of the medium was changed every 4 days.

**Immunoblot analysis**. Cells or brain were lysed in RIPA buffer supplemented with protease inhibitors and phosphatase inhibitors. After clarification by centrifugation at 4^0^C, the lysates were separated on SDS-PAGE in 8% Tris-glycine or 12% Tris-tricine gels (for APP CTFs), and then transferred to nitrocellulose membranes. For the blot of Aβ, lysates were separated on 16% or 20% tricine gel and the membrane after electro-transfer were boiled in PBS in microwave for 2x3min, and cooled down to room temperature. Membranes were blocked in phosphate-buffered saline (PBS) containing 0.1% Tween-200 and 5% BSA, and blotted with indicated antibodies. To specifically blot C99 and Aβ(1–x), C99/Aβ(1–x)–specific antibody 82E1 was used as primary antibody. Membranes were scanned with chemiluminescent imaging system (Tanon 5800) for HRP conjugated secondary antibodies or Odyssey system (LI-COR Biosciences) for fluorescence conjugated secondary antibodies. To detected Aβ in the conditioned medium of HEK293 cells, cells were incubated in DMEM containing 10% FBS overnight after transfection, and then in DMEM without FBS for 24 hours.

**Immunoprecipitation, coimmunoprecipitation and pull-down.** Cells were lysed in lysis buffer containing 20mM Tris (pH7.3), 150mM NaCl, 1mM MgCl2, 1mM EGTA, 1mM EDTA, 1% Triton X100, protease inhibitors and phosphatase inhibitors. After clarification, the supernatants were added to magnetic protein A/G beads with pre-bound antibodies, or to FLAG-conjugated magnetic beads. The proteins bound to beads were eluted with 1xSDS sample buffer for Western blot. For pull-down, the beads after immunoprecipitation were extensively washed in lysis buffer and added to the cell lysates or human serum 1:1000 diluted in PBS (with 0.5% Triton X100).

**Immunofluorescence**. Cells were fixed in 4% paraformaldehyde, washed in PBS, permeabilized in 0.2% Triton X100, blocked in 5% BSA, and stained with primary antibodies diluted in 3% BSA for 1 hour. After washing, cells were stained with Alexa fluorescence labeled secondary antibodies.

**Triton X-100 extraction of primary neuron cells**. Primary neurons in each well of 12-well plate were washed with ice cold PBS, and extracted with 250μl lysis buffer with protease and phosphatase inhibitors at 40C with gentle shaking for 20minutes. The extracts with insoluble cell debris were saved for Western blot after addition of 5×SDS sample buffer and brief sonication. The wells were washed 5 times with lysis buffer before addition of 2xSDS sample buffer. The insoluble proteins were scraped off the well with rubber policeman. For immunofluorescence of the insoluble proteins, cells after extraction were fixed in 4% paraformaldehyde, extensively washed, and blocked in 5% BSA.

**ELISA**. Aβ1–42, and Aβ1–40 in mouse brains were measured using Colorimetric ELISA kits according to the manufacturer’s instructions. Briefly, half brains were weighed and homogenized 5 M guanidine buffer (pH 8.0) to solubilize all Aβ. The homogenates were gently rotated on a rotator at room temperature for 4 hours, diluted 1:10 in PBS, and clarified by centrifugation at 16,000g at 4^0^C for 20min. The supernatants were further 1:5 diluted before ELISA analysis.

***In vitro* Aβ degradations**. For *in vitro* IDE-mediated Aβ degradation, 800nM recombinant human IDE was mixed with 300nM synthetic Aβ1-42 in the presence and absence of 300nM purified recombinant hCLU in PBS containing 5% BSA, and incubated at 37^0^C for the indicated times.  For Aβ degradation by BACE2, 800nM recombinant BACE2, 300nM synthetic Aβ1-42 and 5% BSA were incubated in sodium acetate buffer (pH=4.5) in the presence and absence of 300nM purified recombinant hCLU at 37^0^C for the indicated times.

**Assessment of DNase activity of CSF**. 1μl of 30ng/μl pcDNA4-myc-his A plasmid was incubated in 30μl PBS or with 2pg/ml DNase-I diluted in DNase-I reaction buffer or with 30μl CSF at 37℃ for 24 hours. Plasmids was separated on 1% agarose gel and visualized by EtBr.

**Heparin-Manganese fractionation.** The protocol was as previously described^5^. Briefly, final concentrations of 0.9M MnCl_2_ and 0.205USP U/ml heparin were added to serum, conditioned medium or rhCLU solution, and incubated on ice for 30min. After centrifugation at 12,000g at 4^0^C for 20min. to precipitate the low-density fraction (LF), the supernatant was mixed with 2.4M MnCl_2_ stock solution to bring up MnCl_2_ concentration by 0.218M and with dextran sulfate (15kd) to a final concentration of 0.78%. After incubation on ice for 30min. and centrifugation as before, the supernatant was saved as PF, and the pellet was saved as LF.

**Statistical analyses** were performed using GraphPad Prism 5.0. Results are expressed as mean ± SEM. A 2-tailed Student’s t test was used to analyze the difference between 2 groups. P value less than 0.05 was considered statistically significant. Diagnostic accuracy of SF CLU was assessed from the area under the receiver operative characteristic curve (AUROC) and its 95% confidence intervals (CIs). The AUROC and its 95% confidence intervals CIs were used to evaluate the discriminative ability of SF CLU in identifying dementia patients.

**Supplementary Figures**

**
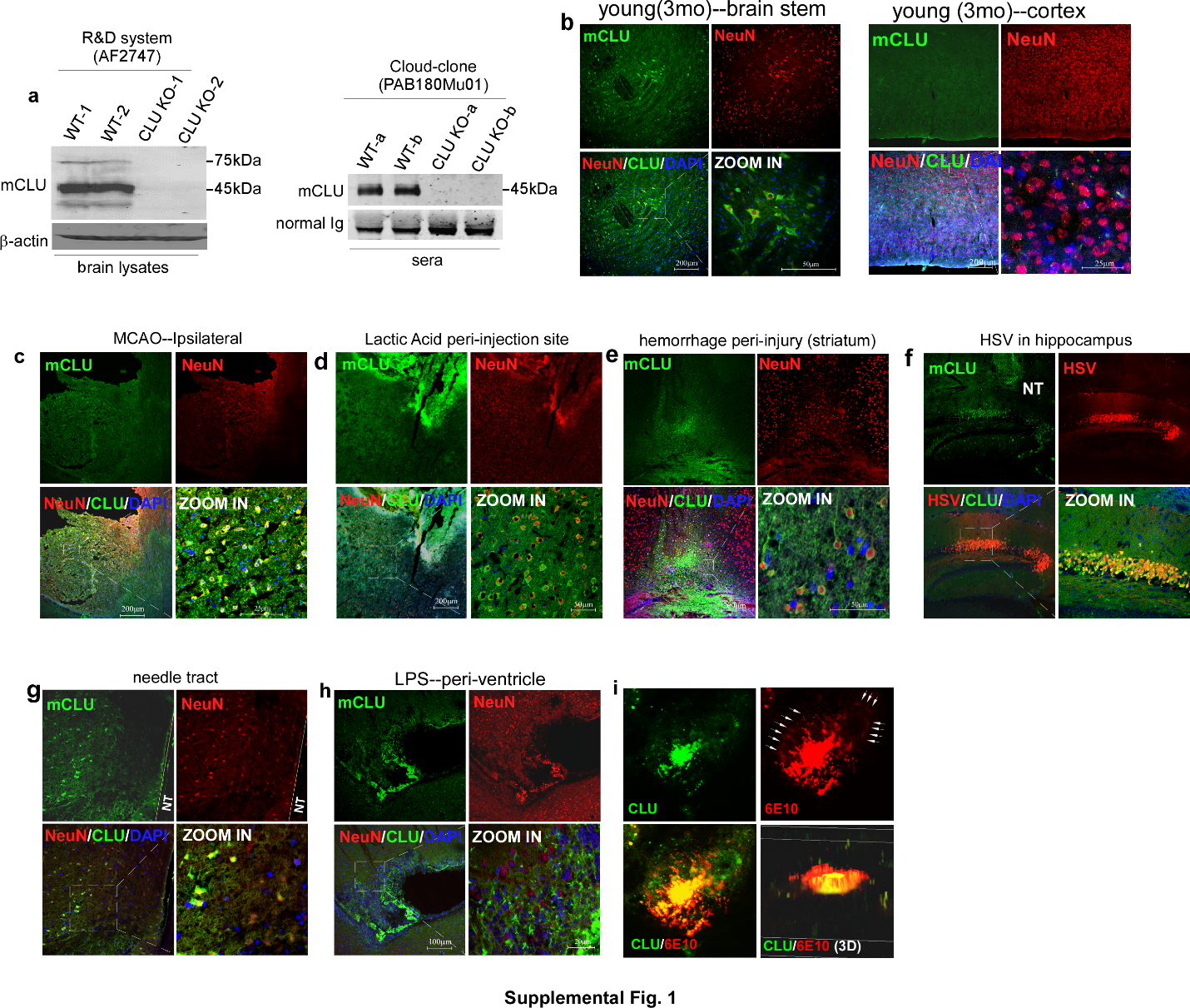
**

**Supplemental Figure 1.** CLU upregulates intraneuronally and extracellularly upon AD risks. (a) antibodies against mouse CLU (mCLU) were validated using CLU KO mouse brains or sera. Brains of mice subject to the following conditions were co-stained with mCLU antibodies and the neuron marker NeuN. (b-h) brains slices of 3-month-old WT mice (b), MCAO-treated stroke mice (c), lactic acid injected mice (d), brain hemorrhage mice (e), HSV1 injected mice (f), needle tracts (g) , and LPS injected mice (h) were co-stained for CLU and neuronal marker NeuN. (i) CLU resides in the dense core of dense-core NPs. The brain slices of 12-month-old *APP/PS1ΔE9* mice were co-stained with with anti-CLU and anti-Aβ antibody (6E10). The arrow heads indicate the halo Aβ staining surrounding the dense core in the center (n=2-3).

**
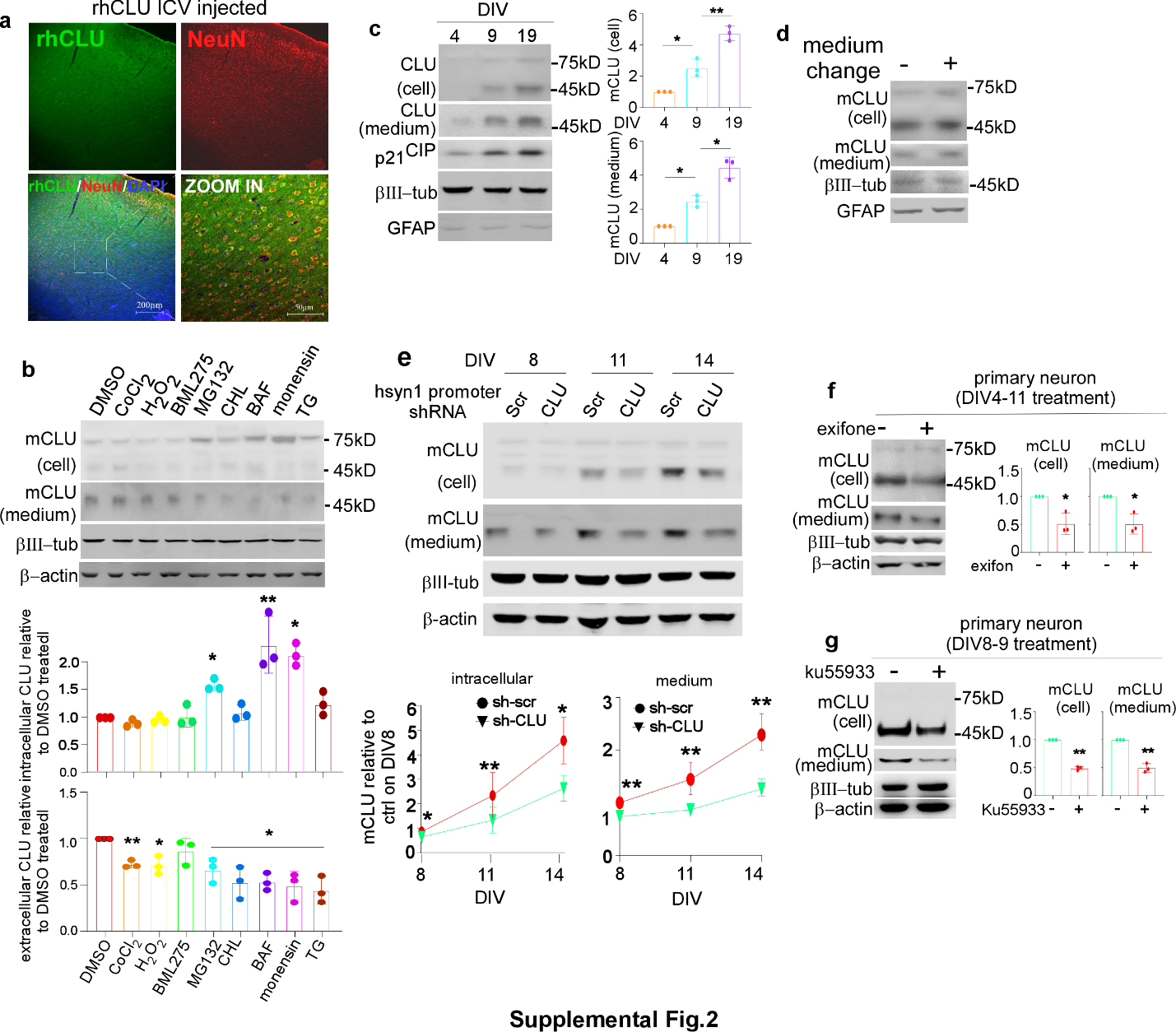
**

**Supplemental Fig. 2**. Neurons uptake CLU and endogenously synthesize CLU. (a) purified recombinant hCLU (rhCLU) was ICV injected, and the brains were co-stained for hCLU and NeuN (n=3). (b) PN of wild-type mice were treated with a variety of stress inducers and blotted for mCLU in cells and in conditioned media. βIII-tubulin and β-actin were used as internal standards. None of these treatments increased mouse endogenous CLU (mCLU) in both cells and the conditioned media. In response to MG132 (a proteasome inhibitor), monensin (an ionophore to increase organellar luminal pH and arrest secretory proteins in the trans-Golgi network), and especially bafilomycin A1 (BAF, to increase organellar luminal pH by inhibiting v-ATPase) treatments, mCLU was significantly increased in cells and decreased in the media. It was previously shown that MG132 suppressed intracellular mCLU in the PN^1^. While this is true for secreted mature mCLU, the increased intracellular mCLU in response to MG132, monensin, and BAF treatments was mostly the ~70kDa form, which is the immature form of mCLU that is to be cleaved in the middle before maturation and secretion. The accumulation of immature hCLU due to mutations in hCLU has been reported for AD patients^2^. n=3 repeats. TG：ER stress inducer thapsigargin. (c) N of wild-type mice were cultured for the indicated times and CLU in cells and conditioned media were blotted. Senescence was indicated by upregulated p21^CIP^. The detection of GFAP suggested contaminant astrocytes that did not grow over time. n=3 repeats. (d) PN were cultured to DIV18 with (+) or without (-) complete medium change. PN and conditioned media were harvested for mCLU detection and quantification. n=3 repeats. (e) PN were infected with AAV9 expressing scrambled *shRNA* (Scr) or shRNA against *mCLU* (CLU) by the neuron-specific *Syn1* promoter on DIV3. PN and conditioned media were collected on DIV8, 11, and 14 to determine the relative amounts of mCLU by Western blot. The time course curve derived from 3 independent experiments were plotted. n=3 repeats. (f) Primary neurons were treated with exifone (2μM) on DIV4, and Neurons and conditioned media were harvested for quantification by Western blot on DIV11. n=3. (g) Primary neurons were treated with ATM kinase inhibitor Ku-55933 (10μM) on DIV8 for 24 hours, mCLU in neurons and conditioned media Western-blotted for quantification. n=3 repeats. *: *p*<0.05, **: *p*<0.01.


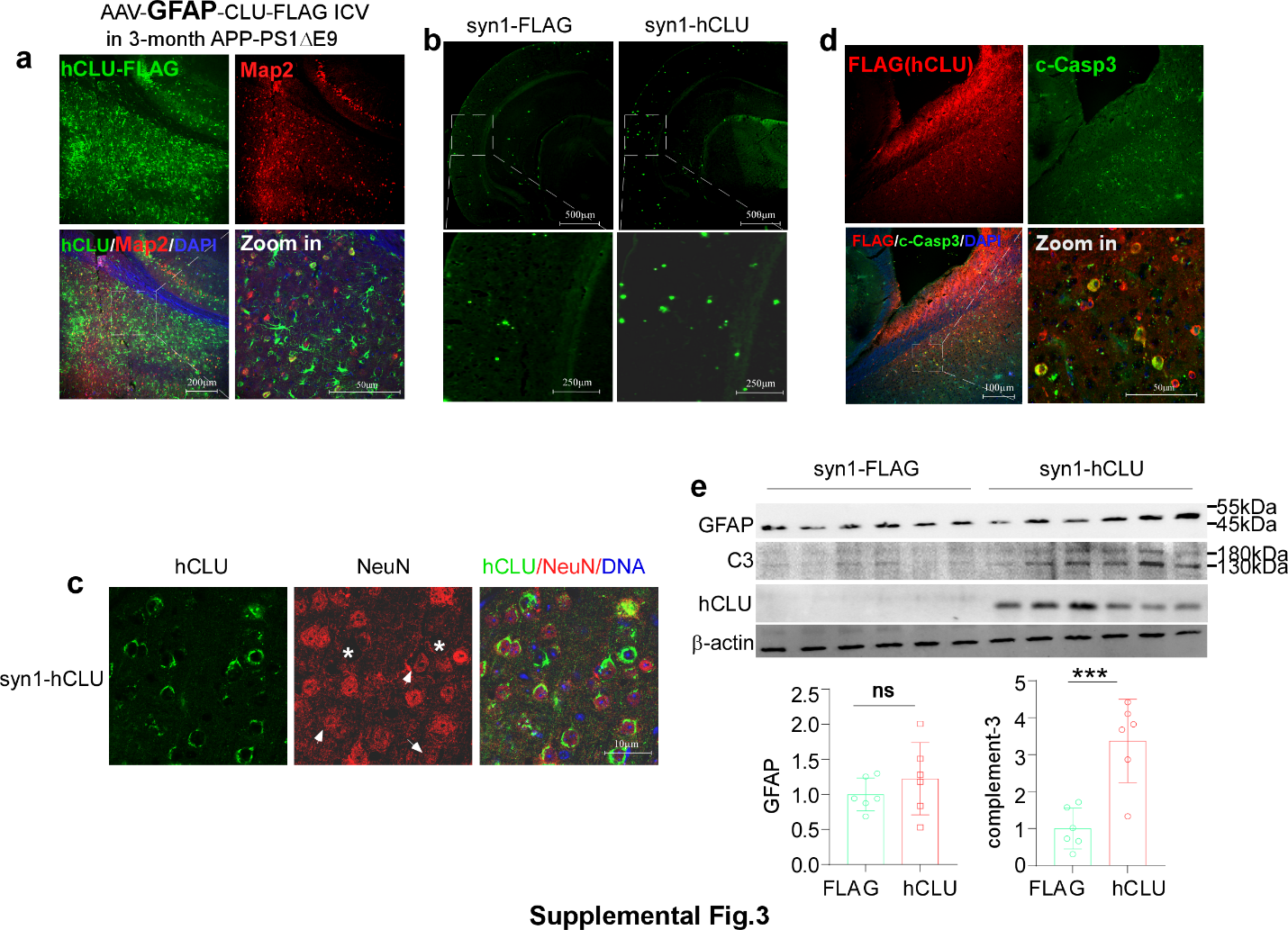


**Supplemental Fig. 3.** The upregulation of Neuronal CLU aggravates AD pathologies in AD model mice. (a) AAV-PHP.EB expressing hCLU by the astrocytic *GFAP* promoter was ICV injected into 5-month-old *APP/PS1ΔE9* mice, and the brains were stained for hCLU and neuron marker NeuN 30 days post injection. n=3. (b) Brains of *APP/PS1ΔE9* mice expressing neuronal hCLU (syn1-hCLU) or control FLAG (syn1-FLAG) were stained with thioflavin-S for NPs. A representative image showing different average sizes of NPs in the two groups. (c) A representative image showing that in neurons with highly expressed hCLU, the neuronal marker NeuN staining was reduced (arrow) or even abolished (*). (d) A representative image showing the expression of cleaved active caspase-3 (c-Casp3) in neurons with high hCLU expression. (e) Brains of *APP/PS1ΔE9* mice with neuronal expression of hCLU (n=6 mice) or control FLAG (n=6 mice) were subjected to Western blot for astrocyte marker GFAP and A1 astrocyte marker complement-3(C3). ***: p<0.001


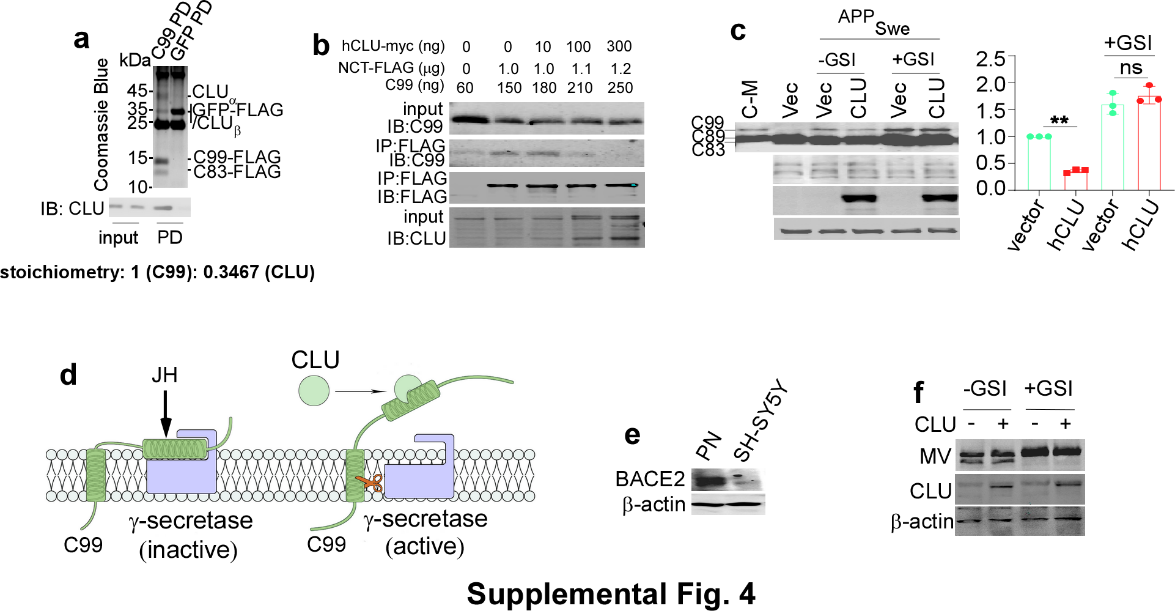


**Supplemental Fig. 4.** CLU increases Aβ generation by disinhibiting γ-cleavage of C99. (a) C99 is a strong CLU binding protein. C99-FLAG was overexpressed in HEK293 cells and immunoprecipitated by FLAG-beads. As C99 can be cleaved by endogenous α-secretases, the APP C-terminus fragment C83 that only weakly binds CLU was also precipitated. The precipitates were used to pull-down (PD) CLU from human serum. GFP-FLAG was used as the control bait protein. The pulled-down proteins were separated on SDS-PAGE and stained by Coomassie blue. CLU consists of two chains derived from the cleavage of immature CLU in the middle and interconnected by disulfide bonds that break upon boiling in the presence of reducing reagents. The N-terminal fragment of CLU is named β-chain and the C-terminal fragment α-chain. The binding stoichiometry was calculated by comparing the ratios of band intensities to molecular weights. (b) hCLU competed with nicastrin (NCT) for C99 binding. Nicastrin-FLAG, non-tagged C99 and increasing Myc-tagged hCLU were co-expressed in HEK293 cells for coimmunoprecipitation (coIP) of nicastrin and C99. As hCLU significantly reduced C99 and to achieve equal expression of C99 for the coIP experiment to compare binding intensities, the amounts of plasmids transfected to cells were adjusted. C99, nicastrin and hCLU were detected using C20 antibody, 9E10 (Myc) and FLAG antibodies, respectively. (c) Overexpressed CLU enhanced γ-cleavage of C99. APPswe was co-expressed with hCLU in PC12 cells and the cells were treated with or without γ-secretase inhibitor (GSI). C-terminal fragments of APP in these cells were blotted with C20 antibody recognizing the last 20 residues of APP and CTFs. CTF markers (C-M) were loaded for the reference of the gel mobilities of CTFs. n=3 repeats. (d) Proposed model of CLU-activated γ-cleavage of C99. JH, juxtamembrane helix. In the absence of CLU, the JH domain of C99 binds to and inhibits γ-secretase. Upon the binding of CLU to the JH of C99, JH dissociates from the γ-secretase, and the γ-cleavage of C99 is activated. (e) BACE2 is highly expressed in primary neurons (PN) compared to cell lines such as SH-SY5Y. BACE2 in the PN and SH-SY5Y lysates were Western blotted. β-actin was used as equal loading control. (f) The MV fragment of Notch that can be directly cleaved by γ-secretase was co-expressed with CLU and treated with or without γ-secretase inhibitor (GSI). MV and the γ-secretase cleaved C-terminal fragment was detected by Myc tag fused to the C-terminus of MV. *: *p*<0.05, **: *p*<0.01, ***: *p*<0.001.


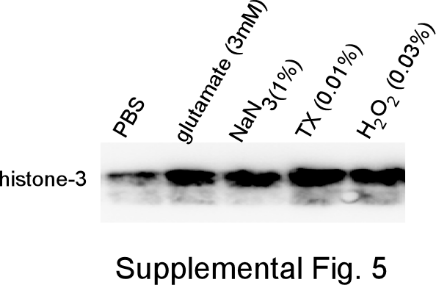


**Supplemental Fig. 5**. Artificially induced neuronal apoptosis or oncosis increases triton x-100 insoluble histones.


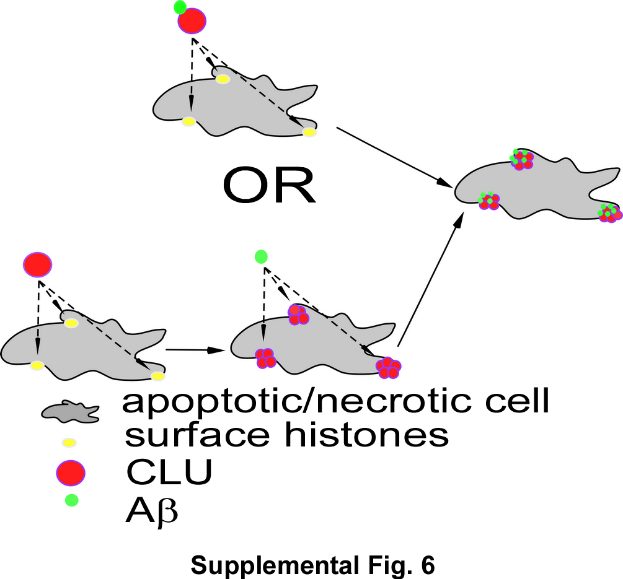


**Supplemental Fig. 6.** Proposed model of CLU dependent Aβ deposition on surface histones in apoptotic cells. Aβ with binding CLU attaches to histones exposed on the surface of apoptotic or necrotic cells that are not cleared by immune/clearance system. Alternatively, CLU attaches to the surface histones independently of binding Aβ, and CLU further recruits soluble Aβ to the sites of dead/dying cells.


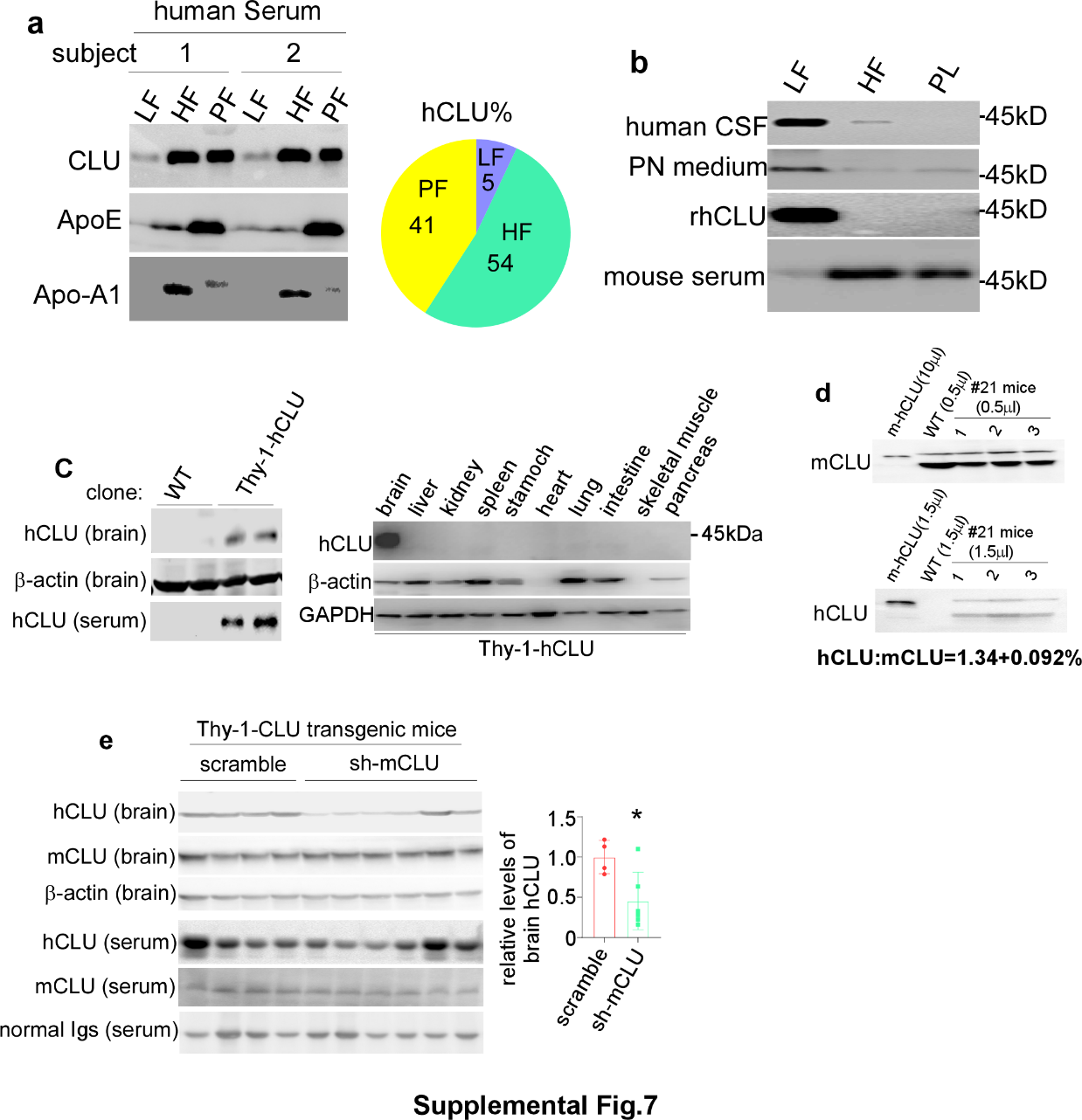


**Supplemental Fig. 7.** Potentials of CLU for clinical applications. (a) Human serum samples were heparin-manganese fractionated. CLU co-precipitated with LDL (LF fraction), HDL (HF fraction) and in the post-lipoprotein fraction (PF) was blotted and quantified. Parts-of-whole chart illustrates the portion of CLU in each fraction. ApoA1 was blotted as a marker for HDL. n=6. (b) Human CSF, conditioned media of PN culture, recombinant hCLU in PBS, and mouse sera were subjected to heparin-manganese fractionation even though there is no LDL or HDL in the samples except mouse sera. Human or mouse CLUs were blotted with their respective antibodies. n=3-6 for each kind of sample. (c) Brains and sera of *Thy-1-CLU* transgenic mice were blotted for hCLU and compared with WT mice. hCLU was clearly detectable only in the brain and serum but not other tissues. (d) In the brains of *Thy-1-hCLU* transgenic mice, hCLU protein was only 1.34±0.092% that of endogenous mCLU. A hCLU and mCLU chimera (h-mCLU) containing the full epitopes of the hCLU antibody and the mCLU antibody were used as the comparison intermediate to quantify the ratio of hCLU to mCLU in the #21 Thy-1-hCLU transgenic mice. A wild-type (WT) mouse was added to demonstrate the specificity of hCLU antibody. For comparison, different amounts of samples and the chimera were loaded to achieve comparable hCLU, mCLU and chimera band intensities. (e) CLU as the target for dementia diagnosis and Aβ suppression. Peripheral suppression of *mCLU* in #21 *Thy-1-hCLU* transgenic mice significantly reduced brain hCLU. AAV8 expressing control *shRNA* (scramble, n=4) or *shRNA* against *mCLU* (sh-mCLU, n=6) were intraperitoneally injected into #21 *Thy-1-hCLU* transgenic mice. 4 weeks post injection, mouse brains and sera were collected for the Western blot and quantification of hCLU and mCLU. β-actin and normal IgG were used as the loading control for brain lysates and sera, respectively. *: *p*<0.05.


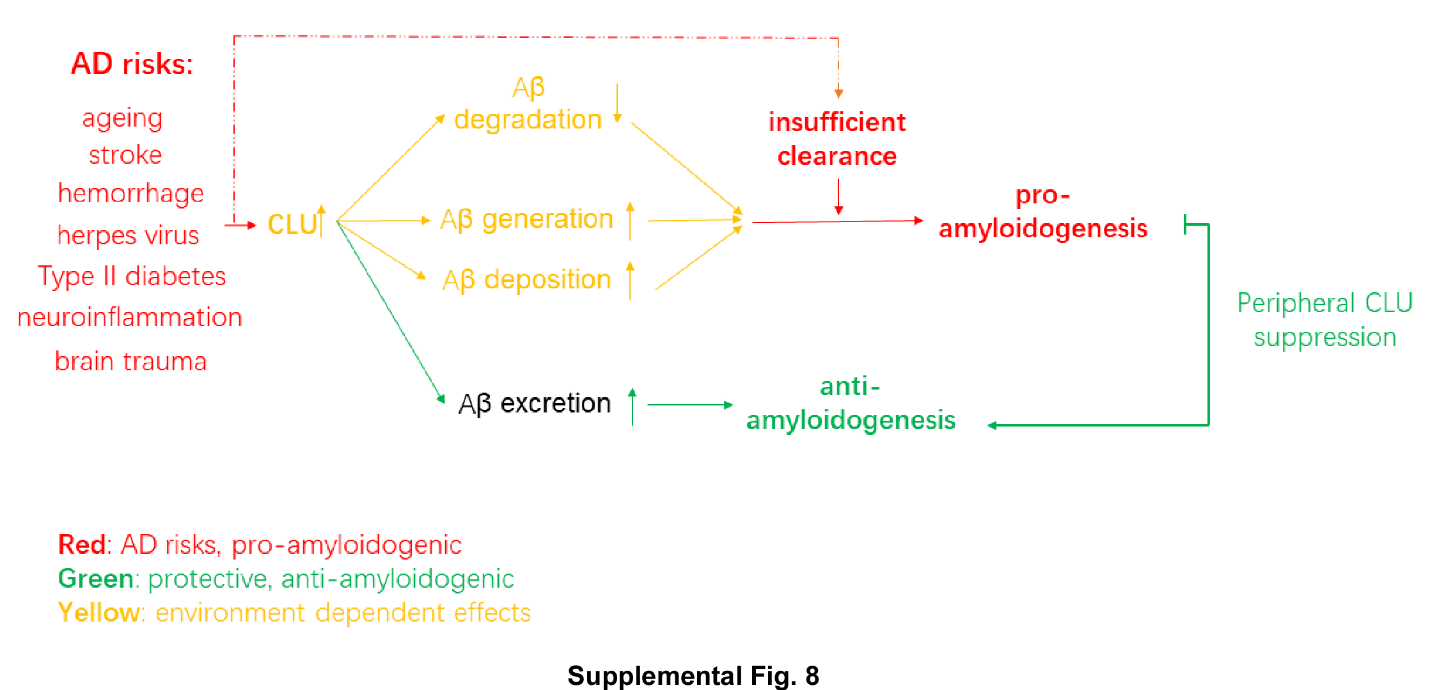


**Supplementary Fig. 8**. Schematic chart showing the proposed mechanism by which CLU transduces AD risk signals to amyloidogenesis, and the rationale on how peripheral CLU suppression may reduce brain Aβ. Upon the risks of AD, brain CLU increases both extracellularly and intraneuronally. The increased CLU may prevent Aβ degradation, and enhance Aβ generation and deposition at the site of cell death. These functions might be meant to prevent the diffusion of toxic components of dead/dying cells, like CLU does in the periphery^6^. Additionally, CLU could also promote the excretion of excessive Aβ from the brain to the periphery^7^. However, as most AD risks also elicit immune responses, and compromise the clearance of dead/dying cells, these cells with binding CLU and Aβ accumulates in the brain, which induces further inflammatory reactions and further Aβ deposition to generate NP. By peripheral CLU suppression, the outflow of CLU and its binding Aβ is accelerated, which prevents the accumulation of CLU and Aβ in the brain.

**Supplementary table-S1**: Mass spectrometry identification of insoluble proteins after Triton X-100 extraction of primary neurons. The presence of these proteins in NPs was reported in shown references


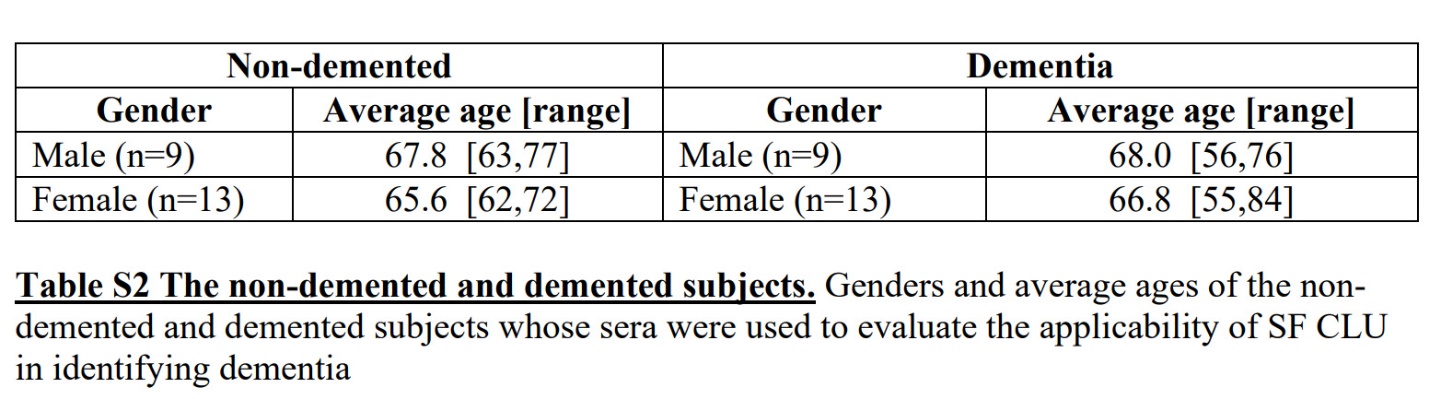


**Extended Note 1**: Clusterin (CLU) is a senescence-associated secretory phenotype protein (SASP)^8^ that is secreted soon after synthesis in cells. The strong physical binding between CLU and Aβ suggests a specific relevance of CLU to AD. CLU is usually induced for protection when the cells are under stress, such as neurons after stroke in adult brains ^9^ and cancer cells during chemotherapy^10^. However, CLU also facilitates Aβ toxicity ^11^ and neuronal death in stroke-challenged neonatal mice ^12^.

*CLU*, ranking 3^rd^ in AD risk genes (http://www.alzgene.org/TopResults.asp), contains several AD-associated SNPs, but the association is much weaker than *ApoE*ε4* that is by far, the strongest genetic risk factor of sporadic AD (SAD) accounting for >95% of total AD cases. However, unlike ApoE4 whose effects are not reproducible in non-ApoE4 carriers because of the different protein sequence ^13^, the AD-associated SNPs in *CLU* with controversial effects on CLU expression^14^ are all in introns. If they mainly modify CLU expression, and considering that CLU is a stress-inducible protein ^10, 12^, stresses might have an even stronger effect than the SNPs on CLU expression. If this is the case, the correlation of CLU, but not the SNPs *per se*, with SAD could have been underestimated. Moreover, the major AD-associated SNP in *CLU* was also correlated with Parkinson’s disease (PD), but the correlation is much better for PD with dementia than without dementia^15^, suggesting a specific interaction between *CLU* and dementia.

CLU is an enigmatic protein and its role in AD has been elusive. Astrocytes are believed to be the major source of *CLU* mRNA, but in the brain, the intracellular CLU protein is either undetectable or exclusively in neurons ^16^ depending on neuron types and age, and it is unknown that if the neuronal CLU is actually, even if in part, endogenously expressed by neurons. Functional studies indicated that CLU prevents spontaneous Aβ aggregation in solution ^17^, facilitates Aβ excretion from the brain to the periphery ^7^, and mediates Aβ clearance through interactions with LRPs ^18^, TREM2 ^19^, and heparan sulfate receptors ^20^. All of these functions would reduce amyloidogenesis. Nonetheless, *CLU* knockout drastically reduces NP in AD mice, suggesting an unidentified pro-amyloidogenic role of CLU that outweighs the anti-amyloidogenic functions ^7, 21, 22^. In *CLU* knockout AD mice, total Aβ is either decreased ^7, 22^ or unaffected ^21^, possibly because of different *APP* mutants overexpressed in these transgenic mice. More confusingly, overexpressing mouse CLU (mCLU) in the astrocytes of neonatal AD mice also reduces NP ^23, 24^. Thus, both the overexpression and depletion of CLU suppress amyloidogenesis. Only when CLU is reduced by ~50% in the *CLU*^+/-^ hemizygous knockout AD mice, is amyloidogenesis enhanced ^23^. However, under what conditions CLU is reduced by 50% in humans is unknown. Moreover, although not being able to enter the brain parenchyma and accumulates in the luminal side of blood vessels, peripherally administered and pre-lipidated CLU reduces Aβ load in an AD model mouse^25^. CLU in the plasma of mice after physical excise suppresses inflammatory genes in the brains of plasma-receiving AD mice^26^. However, CLU upregulation in the blood is also associated with a number of AD risks such as obesity, diabetes, and cardiovascular diseases^27, 28^. Additionally, CLU is also involved in tauopathy, another hallmark of AD. CLU could be both pro- and anti-tau pathology^29, 30^. As CLU is either within the luminal space of secretory pathways (organelles and vesicles) or extracellular, and tau is within the cytoplasm that is segregated with CLU by the organellar membranes, more likely, tau could interact with the minor isoform of CLU that does not enter the secretory pathway.

**Extended Note 2:** Aβ_1-42_ and CLU in human CSF are <2ng/ml (~0.5nM)^31^ and ~20μg/ml (~280nM) ^32^, respectively, CLU is in much excess relative to Aβ_1-42_, although other CLU-binding proteins may compete with Aβ for CLU binding.

**Extended Note 3:** Depending on different *APP* mutants overexpressed in transgenic mice, *CLU* knockout may ^7, 22^ or may not ^21^ suppress Aβ, but always decreases NP.

**Extended Note 4:** Since peripheral CLU cannot cross blood-brain barrier and is stuck at blood vessels ^25, 26^, but CLU in the brain facilitates Aβ excretion to the periphery ^7^, we postulated that suppressing peripheral CLU may reduce CLU and Aβ in the brain by promoting the excretion of CLU and its binding Aβ. Our data indicated that peripheral suppression of CLU did reduce Aβ in the brains. The mechanism underlying this observation however, remains to be investigated. The reduction of brain CLU and Aβ is unlikely a consequence of CLU flow down a gradient, because plasma CLU is several fold higher than CLU in CSF. Presumably, the reduction of peripheral CLU releases and activates receptors required for CLU transcytosis across the blood-brain barrier. Peripheral knockdown of CLU by reagents such as Custirsen (an antisense oligo against *hCLU* to enhance chemotherapy, phase-III clinical trial tested) could be a valid strategy for AD prevention upon the occurrence of AD-risks.

The reduction of brain CLU and Aβ by peripheral CLU suppression appears to be inconsistent with previous studies showing that administering pre-lipidated CLU into the blood could reduce brain NP^25^ and that increased CLU in the blood after physical exercises inhibits the expressions of neuroinflammatory genes^26^. The inconsistence could be due to the different status of CLU lipidation. CLU is an apolipoprotein and is therefore also named apolipoprotein J (ApoJ). In our human- and mouse-based results, more than 50% of CLU in the sera co-fractionated with HDL, but the neuron-generated CLU and CLU in dementia patients are significantly increased in the post-lipoprotein fraction. The non-lipidated CLU, either generated in the brain or in the periphery, might be the involved in amyloidogenesis.

**Extended Discussion:** AD is a neurodegenerative disease with heterogeneous causes. Some geriatric conditions impose high risk of AD development. However, as many AD patients do not have a history of these conditions, milder, containable and asymptomatic forms of these conditions, such as lacunar infarction vs. stroke, may contribute to AD as well. The asymptomatic and symptomatic risks may share similar consequences at the molecular level and finally result in amyloidogenesis and AD. Identifying molecules responding to AD risks and closely related to AD pathologies would greatly benefit AD prognosis and prevention. Molecules acting under all or multiple AD risks are warranted targets, and for clinical applications, they should be non-essential to viability and health.

Our data demonstrated that extracellular and neuronal CLU increase under almost all major AD risks in adult mice. The neuronal CLU under stresses could be from different sources. It could be due to increased CLU expression in neurons, which was observed in aged primary neurons, or the uptake of extracellular CLU secreted by astrocytes, which was evident by the endocytosis of rhCLU into neurons in mouse brains, or the mix of both ways. Another possible cause of increased neuronal CLU is the upregulation of the alternatively spliced CLU isoform (nCLU) that is non-secreted and cytoplasmic. However, the cytoplasmic CLU lower than the immature CLU and higher than the mature CLU on Western blot (antibodies used are against the entire protein or the entire α-chain that is the C-terminal half) was not clearly detected in mouse brain, its contribution to total CLU may not be substantial. Regardless of the source, CLU would enter the secretory pathway, or the luminal side of organelles including ER, Golgi, endosomes or the lysosome, where it encounters the luminal/extracellular domain of APP containing the JH motif and the α-, β-cleavage sites to regulate APP processing.

Although it has been well established that *CLU* gene depletion strongly suppresses NPs, indicating a requirement of CLU for amyloidogenesis *in vivo*, the underlying mechanism remains unknown. On the contrary, some anti-amyloidogenesis functions of CLU have been proposed. In this and previous reports, we showed that CLU inhibited Aβ degradation and enhanced both β- and γ-cleavages of APP to produce more Aβ. By collaborating with exposed histones on apoptotic cells, CLU also facilitated insoluble Aβ deposition to the dead/dying cells *in vitro*. Therefore, CLU is involved in the whole process of Aβ metabolism through specific bindings.

The apoptotic cells in the primary neuron culture of wild-type mouse form insoluble aggregates rich of β-sheet structures, and the protein contents of these apoptotic cell-derived aggregates *in vitro* largely overlapped with those of NP despite of the absence of Aβ. The insoluble aggregates in apoptotic cells rich of CLU were also detected in stroke-challenged wild-type mouse brains. Aβ is not necessary for the formation of these aggregates, instead, it attaches to these aggregates with the help of CLU. Hence, the apoptotic cells may serve as the seeds to nucleate NP, and CLU is indispensable for efficient nucleation. The “NP seed” model explains why NPs adopt round morphology instead of amorphous random aggregates and usually start in some certain regions of the brain. In the healthy brain, the immune system may quickly clear the apoptotic cells to deplete the seeds. With its functions in activating Aβ phagocytosis ^18-20^ and promoting Aβ excretion from the brain ^7^, CLU in the NP or on the apoptotic cells may be meant for efficient clearance of the cell debris by immune cells nearby and for the reduction of excessive Aβ in the brains. However, upon AD risks to impair the clearance system or to generate overwhelmingly too many apoptotic cells, the residual dead/dying cells, as well as CLU and Aβ attaching to them may persist and further recruit CLU and Aβ for amyloidogenesis, which is the initiation of NP (Fig. S7). The requirement of environmental change for NP formation also explains why in familial AD and AD model mice where Aβ is robustly expressed, it still requires decades and 6-12 months, respectively, for NP to extensively accumulate.

**Supplemental references**

1. Longa, E.Z., Weinstein, P.R., Carlson, S. & Cummins, R. Reversible middle cerebral artery occlusion without craniectomy in rats. *Stroke* **20**, 84-91 (1989).

2. Wang, Z. *et al.* BACE2, a conditional beta-secretase, contributes to Alzheimer's disease pathogenesis. *JCI Insight* **4** (2019).

3. Crawley, J.N. Exploratory behavior models of anxiety in mice. *Neurosci Biobehav Rev* **9**, 37-44 (1985).

4. Hughes, R.N. The value of spontaneous alternation behavior (SAB) as a test of retention in pharmacological investigations of memory. *Neurosci Biobehav Rev* **28**, 497-505 (2004).

5. Marmillot, P. *et al.* Long-term ethanol consumption impairs reverse cholesterol transport function of high-density lipoproteins by depleting high-density lipoprotein sphingomyelin both in rats and in humans. *Metabolism* **56**, 947-953 (2007).

6. Cunin, P. *et al.* Clusterin facilitates apoptotic cell clearance and prevents apoptotic cell-induced autoimmune responses. *Cell Death Dis* **7**, e2215 (2016).

7. Wojtas, A.M. *et al.* Loss of clusterin shifts amyloid deposition to the cerebrovasculature via disruption of perivascular drainage pathways. *Proceedings of the National Academy of Sciences of the United States of America* **114**, E6962-E6971 (2017).

8. Basisty, N. *et al.* A proteomic atlas of senescence-associated secretomes for aging biomarker development. *PLoS biology* **18**, e3000599 (2020).

9. Wehrli, P. *et al.* Inhibition of post-ischemic brain injury by clusterin overexpression. *Nature medicine* **7**, 977-979 (2001).

10. Zhang, F. *et al.* Clusterin facilitates stress-induced lipidation of LC3 and autophagosome biogenesis to enhance cancer cell survival. *Nature communications* **5**, 5775 (2014).

11. Killick, R. *et al.* Clusterin regulates beta-amyloid toxicity via Dickkopf-1-driven induction of the wnt-PCP-JNK pathway. *Mol Psychiatry* **19**, 88-98 (2014).

12. Han, B.H. *et al.* Clusterin contributes to caspase-3-independent brain injury following neonatal hypoxia-ischemia. *Nature medicine* **7**, 338-343 (2001).

13. Liu, C.C., Liu, C.C., Kanekiyo, T., Xu, H. & Bu, G. Apolipoprotein E and Alzheimer disease: risk, mechanisms and therapy. *Nature reviews. Neurology* **9**, 106-118 (2013).

14. Foster, E.M., Dangla-Valls, A., Lovestone, S., Ribe, E.M. & Buckley, N.J. Clusterin in Alzheimer's Disease: Mechanisms, Genetics, and Lessons From Other Pathologies. *Frontiers in neuroscience* **13**, 164 (2019).

15. Gao, J., Huang, X., Park, Y., Hollenbeck, A. & Chen, H. An exploratory study on CLU, CR1 and PICALM and Parkinson disease. *PloS one* **6**, e24211 (2011).

16. Pasinetti, G.M., Johnson, S.A., Oda, T., Rozovsky, I. & Finch, C.E. Clusterin (SGP-2): a multifunctional glycoprotein with regional expression in astrocytes and neurons of the adult rat brain. *The Journal of comparative neurology* **339**, 387-400 (1994).

17. Yerbury, J.J. *et al.* The extracellular chaperone clusterin influences amyloid formation and toxicity by interacting with prefibrillar structures. *FASEB journal : official publication of the Federation of American Societies for Experimental Biology* **21**, 2312-2322 (2007).

18. Kounnas, M.Z. *et al.* Identification of glycoprotein 330 as an endocytic receptor for apolipoprotein J/clusterin. *The Journal of biological chemistry* **270**, 13070-13075 (1995).

19. Yeh, F.L., Wang, Y., Tom, I., Gonzalez, L.C. & Sheng, M. TREM2 Binds to Apolipoproteins, Including APOE and CLU/APOJ, and Thereby Facilitates Uptake of Amyloid-Beta by Microglia. *Neuron* **91**, 328-340 (2016).

20. Itakura, E., Chiba, M., Murata, T. & Matsuura, A. Heparan sulfate is a clearance receptor for aberrant extracellular proteins. *The Journal of cell biology* **219** (2020).

21. DeMattos, R.B. *et al.* Clusterin promotes amyloid plaque formation and is critical for neuritic toxicity in a mouse model of Alzheimer's disease. *Proc Natl Acad Sci U S A* **99**, 10843-10848 (2002).

22. Oh, S.B. *et al.* Clusterin contributes to early stage of Alzheimer's disease pathogenesis. *Brain pathology* **29**, 217-231 (2019).

23. Wojtas, A.M. *et al.* Astrocyte-derived clusterin suppresses amyloid formation in vivo. *Molecular neurodegeneration* **15**, 71 (2020).

24. Chen, F. *et al.* Clusterin secreted from astrocyte promotes excitatory synaptic transmission and ameliorates Alzheimer's disease neuropathology. *Molecular neurodegeneration* **16**, 5 (2021).

25. de Retana, S.F. *et al.* Peripheral administration of human recombinant ApoJ/clusterin modulates brain beta-amyloid levels in APP23 mice. *Alzheimers Res Ther* **11**, 42 (2019).

26. De Miguel, Z. *et al.* Exercise plasma boosts memory and dampens brain inflammation via clusterin. *Nature* **600**, 494-499 (2021).

27. Bradley, D. *et al.* Clusterin Impairs Hepatic Insulin Sensitivity and Adipocyte Clusterin Associates With Cardiometabolic Risk. *Diabetes Care* **42**, 466-475 (2019).

28. Turkieh, A. *et al.* Expression and Implication of Clusterin in Left Ventricular Remodeling After Myocardial Infarction. *Circ Heart Fail* **11**, e004838 (2018).

29. Yuste-Checa, P. *et al.* The extracellular chaperone Clusterin enhances Tau aggregate seeding in a cellular model. *Nat Commun* **12**, 4863 (2021).

30. Wojtas, A.M. *et al.* Clusterin ameliorates tau pathology in vivo by inhibiting fibril formation. *Acta Neuropathol Commun* **8**, 210 (2020).

31. Andreasen, N. *et al.* Cerebrospinal fluid beta-amyloid(1-42) in Alzheimer disease: differences between early- and late-onset Alzheimer disease and stability during the course of disease. *Arch Neurol* **56**, 673-680 (1999).

32. Deming, Y. *et al.* A potential endophenotype for Alzheimer's disease: cerebrospinal fluid clusterin. *Neurobiology of aging* **37**, 208 e201-208 e209 (2016).
